# Pharmacoepidemiology and clinical characteristics of medication-related osteonecrosis of the jaw

**DOI:** 10.1186/s40902-019-0210-8

**Published:** 2019-07-23

**Authors:** Hyo-Jeong Son, Jin-Woo Kim, Sun-Jong Kim

**Affiliations:** Department of Oral and Maxillofacial Surgery, School of Medicine, Ewha Womans University, Anyangcheon-ro 1071, Yangcheon-gu, Seoul, 158-710 South Korea

**Keywords:** MRONJ, Weight dose deposited, Bisphosphonate, Pharmacoepidemiology

## Abstract

**Background:**

The aim of this study was to investigate clinical and pharmacoepidemiologic characteristics of medication-related osteonecrosis of the jaw.

**Methods:**

The study population is comprised of 86patients who were diagnosed with ONJ at Ewha Womans University Mokdong Hospital from 2008 to 2015. Factors for epidemiologic evaluation were gender, age, location of lesion, and clinical history. The types of bisphosphonates, duration of intake, and the amount of accumulated dose were evaluated for therapeutic response. Clinical symptoms and radiographic images were utilized for the assessment of prognosis.

**Results:**

Among the 86 patients, five were male, whereas 81 were female with mean age of 73.98 (range 45–97). Location of the lesion was in the mandible for 58 patients and maxilla in 25 patients. Three patients had both mandible and maxilla affected. This shows that the mandible is more prone to the formation of ONJ lesions compared to the maxilla. ONJ occurred in 38 cases after extraction, nine cases after implant surgery, six cases were denture use, and spontaneously in 33 cases. Seventy-six patients were taking other drugs aside from drugs indicated for osteoporosis. Most of these patients were diagnosed as osteoporosis, rheumatic arthritis, multiple myeloma, or had a history of cancer therapy. Higher weighted total accumulation doses were significantly associated with poorer prognosis (*P* < 0.05).

**Conclusion:**

Dose, duration, route, and relative potency of bisphosphonates are significantly associated with treatment prognosis of osteonecrosis of the jaw.

## Background

In 2003, the first cases of what has become known as medication-related osteonecrosis of the jaw (MRONJ) were reported by Marx [1]. Initially, osteonecrosis was reported only after treatment with bisphosphonates and referred to as bisphosphonate-related osteonecrosis of the jaw (BRONJ) [2]. Since 2014, the term MRONJ was recommended by the American Association of Oral and Maxillofacial Surgeons (AAOMS) [3]. The change is justified to accommodate the growing number of cases of osteonecrosis that are associated with other antiresorptive and antiangiogenic treatments in patients who have not used bisphosphonates previously [4–7].

MRONJ is an uncommon condition that can occur after exposure to agents but it is now established as clinically significant, which may cause pain and debilitating conditions in patients, significantly affecting their quality of life [8].

MRONJ is found to be more prevalent in patients with high cumulative doses of bisphosphonates or other agents than in those with lower doses [9, 10] and high incidence of MRONJ has been reported when the agents are administered intravenously (IV) than taken intraorally [11–17]. The mechanism of action of bisphosphonates is not yet well understood, but it essentially involves a powerful inhibition of bone resorption as a result of the reduction of osteoclast activity [18].

The risk factors for MRONJ are classified as medication-related factors, local factors, demographic factors, systemic factors, other medication factors [19–26].

This study aims to investigate the pharmacoepidemiology and clinical features of MRONJ.

## Methods

This study was a retrospective study, analyzing the archived materials. All patients involved in the study took panoramic x-rays to rule out the other etiologies and biopsy was also performed. Patients who were diagnosed with MRONJ at the department of Oral and Maxillofacial Surgery in Ewha Womans University Mokdong Hospital from 2008 to 2015 were included in this study.

### Basic demographics of the samples

Factors for epidemiologic evaluation were gender, age, location of lesion, and clinical history (potential local risk event of MRONJ and underlying bone disease). The information on comorbidity is obtained from the medical records or through the interviews of the patients. The types of bisphosphonates, duration of intake, route of administration, and the amount of accumulated dose were evaluated. Clinical symptoms and radiographic images were used to assess the prognosis for therapeutic response.

### MRONJ staging and treatment respond

The staging system of AAOMS, which may reflect the disease manifestations and help the appropriate assessments on patients [8], we followed MRONJ staging system and treatment assessments by AAOMS recommendations. An initial stage 0 is described, in which there is no clinical evidence of necrotic bone, and yet patients present with non-specific symptom or clinical and radiographic findings [3]. All patients were followed up for 12 months. Treatment response was divided into complete, delayed, and none. ‘Complete’ means completely healed state, while ‘delayed’ means down-staged but not completely healed state and ‘none’ being the same staging state even after the treatment for MRONJ.

### Bisphosphonates

The following variables were analyzed for the bisphosphonates: the average administered dose in milligrams (mg), average dose deposited in bone tissue in milligrams (mg), and weighting dose/potency according to the bisphosphonate used. The following equation was applied to determine the average milligrams (mg) deposited in bone tissue.

Average dose (mg) deposited in bone = average dose (mg) administered × deposit rate to the bone/100 (the percentage of the deposit of bisphosphonates is 1% when taken intraorally and 70% when administered intravenously) [27].

Then, the weight of the dose deposited in the bone tissue was adjusted to the relative potency [28] (Table 2) of each bisphosphonate with the following formula [29]:

Weight dose deposited = average deposited in bone × relative potency.

### Statistical analysis

The statistical analysis was performed using Microsoft Excel and Statistical Package for the Social Sciences (SPSS) ver. 17.0. ANCOVA/Ordinal logistic regression was used to evaluate the correlation between actual weight dose deposited according to the MRONJ Clinical Staging, weight dose deposited according to the treatment response, and factors of age, sex, indication, and medical comorbidities were adjusted. Values of the weight dose deposited were applied logarithm for normal distribution.

## Results

The majority of subjects were females with only 5.8% being males. A total of 86 MRONJ patients with mean age of 73.98 years (range, 45–97) were evaluated. The therapeutic indication for bisphosphonate (BP) was osteoporosis in 76 (88.4%) cases, bone metastasis in six (9.6%) cases, and multiple myeloma in four (6.4%) cases.

The staging was performed using AAOMS criteria. Sixty of the patients (69.8%) were in stage 2, while 15 patients (17.4%) in stage 3 and 11 patients (12.8%) in stage 1. Fifty-eight patients (67.4%) presented osteonecrosis in the mandible, while 25 patients (29.1%) in the maxilla, and three patients (3.5%) in both.

It is found that 38 patients (44.2%) recently had dental extractions, nine (10.5%) implant surgery, and six (7.5%) use dentures. In 33 patients (38.4%), osteonecrosis was apparently spontaneous, without any noticeable or obvious factor.

Still, among these patients, 52 patients were found to have hypertension (HTN), 22 patients to have diabetes mellitus (DM), and 15 patients to have cardiovascular disease (CVD). Also, 15 patients have had steroid therapy and 14 patients had radiotherapy but not in head and neck region. Seven patients had mental disorders and five patients had history of thyroid disease. Four patients had asthma and two patients had end-stage renal disease (ESRD). One patient was found to have liver disease.

Thirty-two patients have been treated with BPs for more than 5 years. Twelve patients were exposed to BPs for between 4 and 5 years while three patients for 3–4 years, 19 patients for 2–3 years, and 14 patients for 1–2 years. Patients who had exposure under 1 year were six.

Seventy patients had oral BPs, while ten patients were administered intravenously. Six patients had both oral and intravenous BPs. Forty-four patients took alendronate, while 18 took Ibandronate, 15 risedronate, three pamidronate, and six zoledronate. The clinical characteristics of the patients who were diagnosed with MRONJ mentioned earlier are grouped (Table 1).

**Table 1:** Clinical characteristics of the patients who were diagnosed with MRONJ

Variable		Number [*n* = 86] (%)
Underlying bone disease	Osteoporosis	76 (88.4%)
	Bone metastasis	6 (9.6%)
	Multiple myeloma	4 (6.4%)
MRONJ stage at diagnosis	Stage 1	11 (12.8%)
	Stage 2	60 (69.8%)
	Stage 3	15 (17.4%)
MRONJ location	Maxilla	25 (29.1%)
	Mandible	58 (67.4%)
	Both	3 (3.5%)
Potential risk events	Spontaneous	33 (38.4%)
	Extraction	38 (44.2%)
	Implant surgery	9 (10.5%)
	Denture use	6 (7.0%)
Medical comorbidities	HTN	52
	CVD	15
	DM	22
	Asthma	4
	RA	14
	Steroid	15
	Thyroid	5
	Mental	7
	ESRD	2
	Liver	1
Duration of BPs exposure	1 year	6 (7.0%)
	1~ 2 year	14 (16.3%)
	2~ 3 year	19 (22.1%)
	3~ 4 year	3 (3.5%)
	4~ 5 year	12 (13.9%)
	> 5 year	32 (37.2%)
Types of BPs	Alendronate	44 (51.2%)
	Ibandronate	18 (20.9%)
	Risedronate	15 (17.4%)
	Pamidronate	3 (3.5%)
	Zoledronate	6 (7.0%)
Route of administration	PO	70(81.4%)
	IV	10 (11.6%)
	Both	6 (7.0%)

Since every underlying disease was counted in each patients. Medical comorbidities was over than 86

Following up the 86 patients, 31 patients were found to be completely healed, while 39 patients were down-staged but not completely healed and 16 patients had no changes in staging.

### Bisphosphonates

Despite its greater dosage, relatively lower potency, and reduced volume of absorption, the average dose of bisphosphonates taken intraorally was higher both in the case of using pamidronate (142,750 mg) and alendronate (17,633 mg) and using ibandronate (51.43 mg) and zoledronate (62.67 mg), compared to the mean doses when administered intravenously. After the adjustment of average administered dose and dose deposited in the bone tissue, a smaller deposit was found in oral formulations and among the drugs; the pamidronate presented the highest deposits in the bone tissue. Finally, by weighing the mean dose deposited in bone tissue with the relative potency referenced to each bisphosphonate, among them highest weight dose deposited/1000 is zoledronate (4386.90) and then, risendronate (1972.25) intravenously (Table 2).

**Table 2:** Main characteristics of bisphosphonates (BPs)

Type of BPs	Relative potency	Average dose administered (mg)	Bone absorption rate	Average dose deposited in bone (mg)	Weight dose deposited/1000
Pamidronate	100	142,750	1% (PO)	1427.5	142.75
		300	70% (IV)	210	21
Alendronate	1000	17,633	1% (PO)	176	176
Ibandronate	10,000	6900	1% (PO)	69	690
		51.43	70% (IV)	36	360.01
Risendronate	20,000	9861.25	1% (PO)	98.61	1972.25
Zoledronate	100,000	62.67	70% (IV)	43.87	4386.90

Relative potency, average dose administered, bone absorption rate, average dose deposited in Bone, weight dose deposited/1000 in this study

### Weight dose deposited according to MRONJ clinical stage and treatment response

**Fig. 1:**
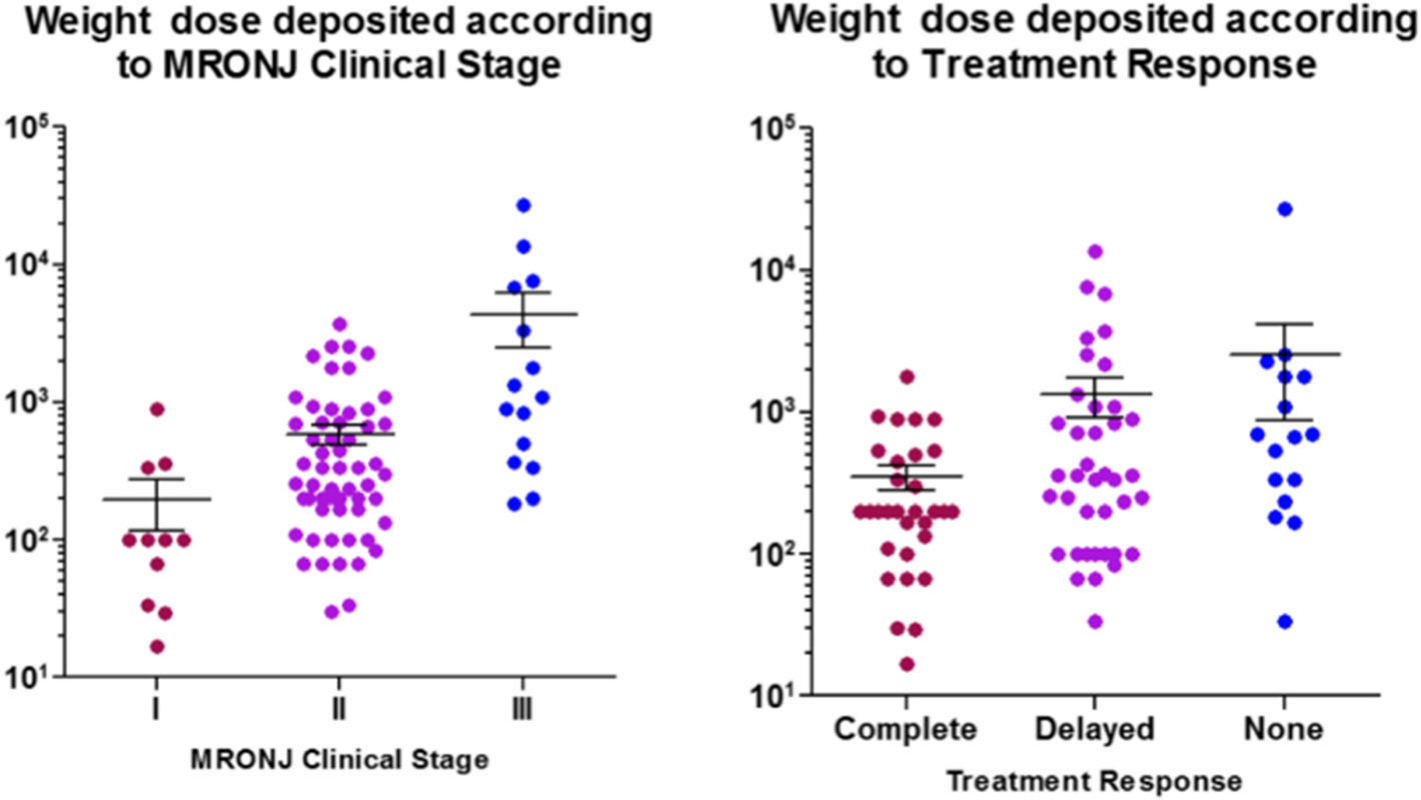
Weight dose deposited according to MRONJ Clinical Stage and Treatment Response. Actual weight dose deposited were calculated and the values were awarded graph

Values of actual weight dose deposited/1000 was calculated and averaged for each stage and stage I was 195.15 (SD260.97), stage 2 was 589.82 (SD746.3), and stage 3 was 4379.89 (SD7317.72). Calculating the logarithm of actual weight dose deposited, Ln (weight dose deposited), stage 1 was 4.43 (SD 1.18), stage 2 was 5.77 (SD 1.11), and stage 3 was 7.28 (SD 1.54). The higher the stage, the higher the weighted total accumulation was found (*P* < 0.001) (Fig. 1, Table 3). When the mean weight dose deposited/1000 value was calculated according to the response to treatment, ‘complete’ had values of 351.18 (SD385.32), while ‘delayed’ had 1328.92 (SD2607.23), and none had 2532.48 (SD6604.07). The higher weighted total accumulation was found as the response to therapy was delayed or none (Fig. 1, Table 3) (*P* < 0.05). In addition, in MRONJ, weighted dose depositions were found to have a significant effect on ONJ staging and treatment response, measured by ordinal logistic regression, with 95% of confidence level (*P* < 0.05) (Table 4).

**Table 3:** Weight dose deposited according to MRONJ Clinical Stage and Treatment Response

	Weight dose deposited (SD)	Ln [weight dose deposited] (SD)	*P*
MRONJ stage			
I	195.15 (260.97)	4.63 (1.18)	< 0.001
II	589.82 (746.43)	5.77 (1.11)	
III	4379.89 (7317.72)	7.28 (1.54)	
Treatment response			
Complete	351.18 (385.32)		< 0.05
Delayed	1328.92 (2607.23)		
None	2532.48 (6604.07)		

ANCOVA adjusted age, sex MRONJ site, medical comorbidities

Results are shown as mean (SD)

Values of the weight dose deposited was applied logarithm for normal distribution

The higher the stage, the higher the weighted total accumulation was found (*P* <  0.001)

Higher weighted total accumulation was found as the response to therapy was delayed or none (*P* <  0.05)

**Table 4:** Weight dose deposited according to MRONJ Clinical Stage and Treatment Response

	Estimates (SE)	95%	CI	Significance
Weight dose deposited according to MRONJ Clinical Stage				
Ln [weighted dose]	1.113 (0.247)	0.63	1.60	< 0.001
Age	− 0.043 (0.030)	− 0.10	0.02	> 0.05
Sex	− 1.033 (1.168)	− 3.32	1.23	> 0.05
MRONJ site	1.635 (1.344)	− 1.00	4.27	> 0.05
Medical comorbidity	0.089 (0.539)	− 0.97	1.15	> 0.05
ONJ stage	5.921 (2.898)	0.24	11.60	< 0.05
Weight dose deposited according to treatment response				
Ln [weighted dose]	0.443 (0.160)	0.13	0.76	< 0.05
Age	0.012 (0.023)	− 0.03	0.06	> 0.05
Sex	0.237 (0.918)	− 1.56	2.04	> 0.05
MRONJ site	− 0.917 (1.135)	− 3.14	1.31	> 0.05
Medical comorbidity	0.476 (0.440)	− 0.39	1.34	> 0.05
Tx. response	4.757 (2.354)	0.14	9.37	< 0.05

Ordinal logistic regression adjusted age, sex MRONJ site, medical comorbidities

Weighted dose depositions were found to have a significant effect on ONJ staging and treatment response, measured by ordinal logistic regression, with 95% of confidence level (*P* < 0.05)

## Discussions

Oral BPs are agents commonly used for osteoporosis and osteopenia, while intravenous BPs may also be used to manage cancer-related conditions, especially for bone metastases of a breast, lung, or prostate primary solid cancer, or lytic lesions developed in patients with multiple myeloma [30–35]. Considering the demographic factors, higher prevalence of ONJ is reported in the female population. This can be explained by the therapeutic indication (breast cancer, osteoporosis) [7].

In this study, female patients were found to account for significantly higher portions than male. Among the underlying diseases, osteoporosis accounted for high portions. It can be due to the female with osteoporosis patients being the majority.

Alendronate (Fosamax®) was found to be the main inducer of oral MRONJ because of its extensive use with other bisphosphonates [36]. Alendronate was most commonly used in this study as well. However, as the use of other bisphosphonates and other drugs gradually increase [37], alendronate will be less blamed for the leading cause of BRONJ associated with oral administration.

Many additional factors have been reported in the literature as being associated with acceleration of the MRONJ [38, 39]. They include the use of corticosteroids, the presence of concomitant diseases, or conditions such as diabetes mellitus. Therefore, the general medical history and medication of the patients should be considered.

As shown in the definition of term MRONJ, MRONJ is only found in the mandible and maxilla, highlighting their unique nature compared with other parts of the skeleton. The jaws are the only bones in the human body that are in frequent contact with the external environments and are subject to repeated microtrauma through the presence of teeth and the forces of mastication. Moreover, the turnover of alveolar bone is tenfold greater than in the long bones. While BPs can decrease this turnover, the alveolar remodeling still remains to be more frequent when compared with the long bones. MRONJ affects the mandible more often than the maxilla, while both jaws involvement is rare [40]. In our study, mandibles were found to be affected more than maxilla as well.

Local trauma that are caused by tooth extractions, local surgery, or ill-fitting dentures are the most important risk factors, which are being consistently reported throughout the literature [12]. It is probably multifactorial, with infection and trauma to the bone or soft tissue playing important roles [41]. In this study, 61.6% patients were found to have local risk factor like tooth extraction (44.2%). Therefore, patients who are at risk of developing MRONJ need to be educated with adverse effects of the medication like BPs and dentist and dental hygienist should guide the patients for good oral hygiene and put efforts to as little oral irritation as possible at the dental field.

Previous studies have shown that the longer the time of exposure to the drug, the greater the likelihood of being affected by MRONJ. Thus, protocols for dental procedures have been reported in patients taking BPs, taking into account the MRONJ risk associated with the duration of drug use [42, 43]. In this study, weighted total accumulation was found to be higher in patients with higher MRONJ clinical stage from stage 1 to stage 3. Also, when treated according to the MRONJ treatment protocol, the higher the patient’s weighted total accumulation, the more delayed the response to treatment. It is suggested that in a pharmaco-pathologic view, the amount of the drug accumulated in the bone due to the average administered dose and the absorption rate of the drug, and relative potency of the drug, will be more important than the absolute period of exposure to the drug.

Since 2003, when the adverse effects that bisphosphonates administration on the jaw was recognized, numerous evidence-based case reports have been published in the international literature [44–46] .Zoledronic acid is, by far, the foremost intravenous drug associated with osteonecrosis [47, 48]. Zoledronic acid, administered intravenously over intraorally, has a high accumulation in bone and a relatively high relative potency [27]. The larger the accumulation of the drug in the bone and the greater the relative potency, the larger the weight dose deposited. As found in this study, when administered intravenously, weight dose deposited dose of zoledronate is about 25 times as much as that of the zoledronate when given intraorally.

Physicians believe the benefit of BPs and other drugs used for anti-resorptive and anti-angiogenic therapies outweighs the risk of development of MRONJ in the settings of both osteoporosis and oncology [49, 50]. However, since the risks of ONJ may increase with the amount accumulated in the bone and the relative potency increase, physicians should consider the likelihood and risk of MRONJ when selecting and prescribing the medications in osteoporosis or oncology.

There are still controversies on the benefit of temporary drug holidays with bisphosphonates or other drugs inducing MRONJ in patients who are scheduled to receive invasive dental procedures. The increased risk of pathologic fracture during drug holidays [51] must be balanced with the reduced risk development of MRONJ for individual cases and should be discussed in a multidisciplinary manner.

## Conclusion

Cumulative dose of bisphosphonates deposited in hard tissue is significantly associated with clinical staging and treatment response. Before initiating dental procedures, dentists need to evaluate the medical history and reassess the risk of MRONJ proactively. Especially, the medical history of a patient should be gone through for doses used, route of administration, and exposure duration to bisphosphonate or other agents that may increase the risks of MRONJ.

## Abbreviations

AAOMS: American Association of Oral and Maxillofacial Surgeons
BPs: Bisphosphonates
BRONJ: Bisphosphonate-related osteonecrosis of the jaw
CVD: Cardiovascular disease
DM: Diabetes mellitus
ESRD: End-stage renal disease
HTN: Hypertension
MRONJ: Medication-related osteonecrosis of the jaw
ONJ: Osteonecrosis of the jaw
SD: Standard deviation
